# Renal injury is accelerated by global hypoxia-inducible factor 1 alpha deficiency in a mouse model of STZ-induced diabetes

**DOI:** 10.1186/s12902-017-0200-8

**Published:** 2017-08-03

**Authors:** Romana Bohuslavova, Radka Cerychova, Katerina Nepomucka, Gabriela Pavlinkova

**Affiliations:** 1 Laboratory of Molecular Pathogenetics, Institute of Biotechnology CAS, BIOCEV, Center of Excellence, Prumyslova 595, Vestec, 25242 Czechia; 2 Faculty of Science, Charles University, Prague, Czechia

**Keywords:** Diabetic complications, Diabetic nephropathy, Hypoxia, Podocyte, Mouse model

## Abstract

**Background:**

Hypoxia inducible factor 1 (HIF-1) activates protective pathways to counteract hypoxia and prevent tissue damage in conjunction with renal injury. The aim of this study was to evaluate a role of HIF-1 in diabetes-induced kidney damage.

**Methods:**

We used a streptozotocin-induced diabetes mouse model and compared biochemical, histological and molecular parameters associated with kidney damage in *Hif1α* deficient (*Hif1α* ^*+/-*^) and wild-type mice.

**Results:**

We showed that *Hif1*α deficiency accelerated pathological changes in the early stage of DN. Six weeks after diabetes-induction, *Hif1*α deficient mice showed more prominent changes in biochemical serum parameters associated with glomerular injury, increased expression of podocyte damage markers, and loss of podocytes compared to wild-type mice. These results indicate that *Hif1*α deficiency specifically affects podocyte survival in the early phase of DN, resulting in diabetic glomerular injury. In contrast, renal fibrosis was not affected by the global reduction of *Hif1α*, at least not in the early phase of diabetic exposure.

**Conclusions:**

Together our data reveal that HIF-1 has an essential role in the early response to prevent diabetes-induced tissue damage and that impaired HIF-1 signaling results in a faster progression of DN. Although the modulation of HIF-1 activity is a high-priority target for clinical treatments, further study is required to investigate HIF-1 as a potential therapeutic target for the treatment of DN.

**Electronic supplementary material:**

The online version of this article (doi:10.1186/s12902-017-0200-8) contains supplementary material, which is available to authorized users.

## Background

Diabetic nephropathy (DN) is an endemic complication of diabetes and the leading cause of end-stage renal failure. Clinical features of DN are progressive albuminuria, proteinuria, and an eventual reduction in the glomerular filtration rate [1]. The complex progressive histopathological changes associated with DN include mesangial matrix expansion, thickening of basement membranes, glomerular and tubular hypertrophy, podocyte loss, and glomerulosclerosis and tubulointerstitial fibrosis [2]. High glucose is a primary initiating factor of multiple molecular, metabolic, and hemodynamic changes resulting in kidney damage, including intrarenal tissue hypoxia [3]. Tissue hypoxia activates multiple pathways, such as profibrotic growth factors, hemodynamic cytokines (angiotensin II), advanced glycation end products (AGE), and reactive oxygen species (ROS). Thus, both hyperglycemia and hypoxia are major determinators of the chronic complications associated with diabetes.

A master regulator of transcriptional responses to hypoxia is hypoxia inducible factor 1 (HIF-1). HIF-1 has been recently associated with the progression of chronic renal injuries including DN [4–6]. HIF-1 consists of two subunits, HIF-1α, an O_2_-labile subunit, and constitutively expressed HIF-1β [7]. *Hif1α* ^*+/−*^ heterozygote mutants demonstrate impaired responses when challenged with hypoxic conditions after birth [8, 9]. HIF-1 directly regulates the expression of more than 1000 human genes (for review, see [10]). Although the expression of a subset of HIF-1 target genes is induced by hypoxia in most or all cell types, the majority of these genes are induced by hypoxia in a cell type–specific manner. In addition to hypoxia, the HIF-1α subunit activity is regulated by numerous other factors, including growth factors, cytokines, sirtuins, ROS, and intracellular metabolites, even under normoxic conditions [11]. However, the mechanism of HIF-1α stabilization in a hyperglycemic environment is controversial. Hyperglycemia upregulates HIF-1α in the glomeruli of diabetic model mice regardless of the etiology of the diabetes [5, 12]. The activation of HIF-1 in the diabetic kidney may be suboptimal despite profound renal hypoxia, as suggested by a large body of evidence showing that the diabetic milieu deregulates the HIF-1α pathway [13–15]. It remains controversial whether the activation of HIF-1 signaling exerts a beneficial or harmful role in the progression of renal diseases, particularly DN. An indirect approach using YC-1 [3-(5′-hydroxymethyl-2′-furyl)-1-benzyl indazole], a HIF-1 inhibitor, reduced glomerular hypertrophy and AGE-tissue modifications in the type 1 diabetes mouse model [6]. In contrast, an activation of HIF-1α by CoCl_2_ reduced proteinuria and histological markers of kidney injury in an obese type 2 diabetes model [16] and in STZ-induced DN in rats [3].

To provide more insight into the functional role of HIF-1α pathways, we examine the relationship between diabetes-induced kidney injury and the partial deficiency of HIF-1α caused by the global deletion of the *Hif1α* functional allele with a specific focus on the early phase of diabetes-exposure. Together, our data suggest the potential roles of HIF-1α and *Hif1α* genetic variations in the manifestation of DN. Furthermore, our data point out the necessity of optimizing any possible pharmacological inhibition of HIF-1 in therapeutic applications of DN and diabetes-associated pathologies.

## Methods

### Experimental animals

This study was conducted in accordance with the Guide for the Care and Use of Laboratory Animals (NIH Publication No. 85-23, revised 1996). The experimental protocol was approved by the Animal Care and Use Committee of the Institute of Molecular Genetics, CAS. Diabetes was induced in male inbred FVB (*Wt*, strain code 207, Charles River) and *Hif1α* ^*+/−*^ strain on the FVB background, aged 7–9 weeks, by 2 intraperitoneal injections of 100 mg/kg body weight of streptozotocin (STZ; Sigma, St. Louis, MO), as described [17, 18]. Mice were sacrificed after 6 weeks of diabetes at age 15-17 weeks. The *Hif1α* mutants with the *Hif1* ^*atm1Jhu*^ mutant allele [19] were obtained from Prof. Gregg L. Semenza. *Hif1α* ^*+/−*^ mice showed a partial loss of HIF-1α protein expression levels [20, 21]. The *Hif1α* ^*+/−*^ mouse colony was bred and maintained in our laboratory. Offspring of *Wt* x *Hif1α* ^*+/−*^ matings were genotyped by PCR [22], using DNA isolated from tails and amplifying neomycin (*Neo*) and *Hif1α* exon 2 sequences [19]; *Neo* (463-bp) and *Hif1α* (317-bp).

### Biochemical parameters

Blood serum was collected following a 6-h fast (from 7 a.m. to 1 p.m. as recommended by the NIH for mouse metabolic models [23]) and was analyzed using a Beckman Coulter AU480 Chemistry Analyzer (Beckman) according to the manufacturer’s protocol in the Core Facility of Czech Centre for Phenogenomics in Biocev.

### Real-time reverse-transcription PCR (RT-qPCR)

Total RNA was isolated from the renal cortex of diabetic *Wt,* non-diabetic and diabetic *Hif1α* ^*+/−*^(EXP), and from non-diabetic *Wt* (control); the renal medulla was discarded. Following RT, quantitative real-time PCR (qPCR) was performed as described [9]. The relative expression of a target gene was calculated, based on qPCR efficiencies (E) and the quantification cycle (Cq) difference (Δ) of an experimental sample versus control (ratio = (*E*_target_)^ΔCq Hif1a(Mean control – Mean EXP)^/(*E*_Hprt1_)^ΔCq Hprt1(Mean control – Mean EXP)^. RT-qPCR data were analyzed using the GenEX5 program (www.multid.se/genex/genex.html). Primer sequences are presented in Additional file 1: Table S1.

### Western blot

The renal cortexices from the diabetic and non-diabetic kidneys were lysed with protease and phosphatase inhibitors to prevent protein degradation and stored at −80 °C until analysis. Fifty microgram of total protein lysates were denatured, resolved using 10% SDS-PAGE, and transferred to a nitrocellulose membrane, as described in detail previously [18]. The membrane was blocked with 5% dry milk and incubated overnight with rabbit anti-CX43 antibody at 1:6000 (#C6219, Sigma), or anti-VEGFA at 1:200 (#sc-7269; Santa Cruz Biotechnology, TX, USA). After incubation with a horseradish peroxidase–conjugated secondary IgG (Sigma), the blots were developed using the SuperSignal™ West Femto Maximum Sensitivity Substrate (#34095; Thermo Scientific, MI, USA). Chemiluminescent signals were captured using an ImageQuant LAS 4000 Imager (GE Healthcare Bio-Sciences AB, Sweden) and analyzed by ImageJ software (http://imagej.nih.gov/ij/download.html). Ponceau S staining was used as the loading control.

### Histology and immunohistochemistry

To detect tissue modifications and tissue remodeling we used the Periodic acid–Schiff (PAS) staining system (#395B, Sigma, St. Louis, MO) and Trichrome Stain (Masson) Kit (#HT15-1KT, Sigma), respectively. Paraffin sections (8 μm) were dehydrated and used for the both methods. PAS^+^ area was delineated using the Adobe Photoshop CS5.1 program. Quantification of the PAS and collagen positive areas was performed using the threshold tool in the ImageJ program **(** http://imagej.nih.gov/ij/download.html), separating pixels which fall within a desired range of intensity values from those which do not.

Sections (8 μm) for immunohistochemistry were heated in citrate buffer (0.07 M, pH 6.0) for antigen retrieval and blocked with PBS (pH 7.4) with 0.1% Tween®20 (#P9416, Sigma) and 10% normal goat serum (#005-000-121, Jackson Immuno Research Labs). Primary antibodies used: mouse anti-VEGFA 1:50 (#sc-7269, Santa Cruz Biotechnology), rabbit anti-pHH3 1:100 (#06-570, Merck Millipore), rabbit anti-WT1 1:200 (#CA1026, Merck Millipore) and mouse anti-alpha smooth muscle actin (α-SMA) 1:400 (#A2547, Sigma). Secondary antibodies used: Alexa Fluor® 488 and 594 1:400 (#115-545-146 and #111-585-144, resp., Jackson Immuno Research Labs). The sections were counterstained with Hoechst 33,342 (#14533 Sigma) and imaging with confocal microscope (ZEISS LSM 880 NLO)*.* The areas of VEGFA and α-SMA expression, and a number of WT1^+^ podocytes and pHH3^+^ nuclei in the renal cortex were quantified using the ImageJ.

### Statistics

All values are means ± SEM. We used two-way ANOVA to compare differences among experimental groups with genotype and experimental condition (diabetes or no diabetes) as categories. When a significant interaction was detected, the differences between subgroups were further analyzed by post hoc Tukey’s multiple comparison tests; significance assigned at the *P* < 0.05 level (Graph Pad, 2005; Graph Pad, San Diego, CA).

## Results

### Changes in physiological and biochemical parameters after 6 weeks of diabetes

**Fig. 1:**
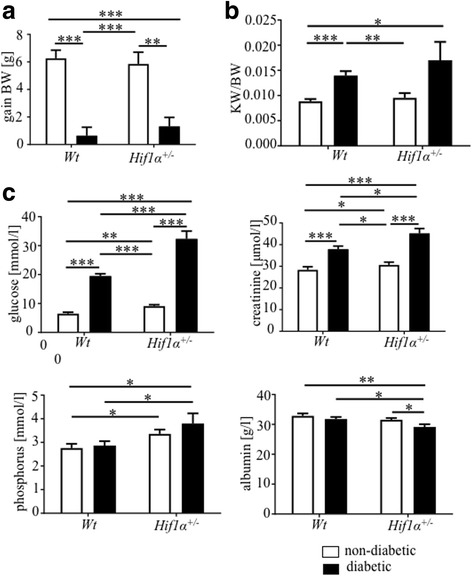
Physiological and biochemical parameters. **a** Body weight gain after 6 weeks from the induction of diabetes. **b** Changes in kidney/body weight ratio (KW/BW) after 6 weeks of diabetes exposure. The values represent means ± SEM (non-diabetic *Wt* (*n* = 9), diabetic *Wt* (*n* = 11), non-diabetic *Hif1α* ^*+/−*^ (*n* = 6) and diabetic *Hif1α* ^*+/−*^ *(n* = 12)). Two-way ANOVA showed significant effect of diabetes in the body weight gain (*P* < 0.0001) and in the KW/BW (*P* = 0.0005). **c** The levels of glucose, creatinine, phosphorus, and albumin in the blood serum of *Wt* and *Hif1α* ^*+/−*^ mice after 6 h fasting and collected at the end of experiment (6 weeks from the induction of diabetes). The values represent means ± SEM (*n* = 8 mice in each group). Statistical significance assessed by two-way ANOVA: genotype effect (creatinine, *P* < 0.001; phosphorus, *P* = 0.0037; albumin, *P* = 0.027); diabetes effect (creatinine, *P* < 0.0001; albumin, *P* = 0.027); and interaction between genotype and diabetes: glucose *P* < 0.0001. *Significant differences by post hoc pairwise comparison tests, *P < 0.05, **P < 0.01, ***P < 0.001

For this study we used the well-established STZ-induced diabetes mouse model on the FVB genetic background [17, 18]. Age-matched wild-type *Hif1α* ^*+/+*^ (*Wt)* and *Hif1α* ^*+/−*^ mice were compared. Body weight gain after 6 weeks was significantly decreased in diabetic mice of both genotypes (Fig. 1a; non-diabetic *Wt* 6.3 ± 0.6 g (*n* = 9), diabetic *Wt* 0.6 ± 0.6 g (*n* = 11), non-diabetic *Hif1α* ^*+/−*^ 5.9 ± 0.9 g (*n* = 6) and diabetic *Hif1α* ^*+/−*^ 1.3 ± 0.7 g (*n* = 12)). In contrast, the kidney weight-to-body weight ratios were increased in diabetics compared to controls (Fig. 1b; non-diabetic *Wt* 0.009 ± 0.0004 g (*n* = 17), diabetic *Wt* 0.014 ± 0.001 g (*n* = 8), non-diabetic *Hif1α* ^*+/−*^ 0.010 ± 0.001 g (*n* = 6) and diabetic *Hif1α* ^*+/−*^ 0.017 ± 0.004 g (*n* = 11)), consistent with diabetic renal hypertrophy phenotype [4, 24].

Increased serum levels of creatinine and phosphorus, and decreased serum levels of albumin are the first markers of kidney damage due to high glucose concentrations [4]. Blood serum was collected from non-diabetic and diabetic *Wt* and *Hif1α* ^*+/−*^ mice after 6 h-fasting. Both *Wt* and *Hif1α* ^*+/−*^ mice developed high levels of hyperglycemia after STZ injections over the 6-week study (Fig. 1c). Interestingly, serum glucose levels were significantly higher in diabetic *Hif1α* ^*+/−*^ compare to diabetic *Wt* mice. Serum phosphorus levels were also slightly higher in non-diabetic *Hif1α* ^*+/−*^ compared to non-diabetic *Wt* mice (Fig. 1c). As serum phosphorus is a cardiovascular risk factor [25], these data correspond with a predisposition of *Hif1α* ^*+/−*^ mutation for endothelial dysfunction and cardiovascular disease [8, 9, 18]. Phosphorus and creatinine levels were significantly increased, whereas the levels of albumin were significantly reduced in diabetic *Hif1α* ^*+/−*^ compared to diabetic *Wt* mice (Fig. 1c).

### Tissue modification and remodeling in the renal cortex

**Fig. 2:**
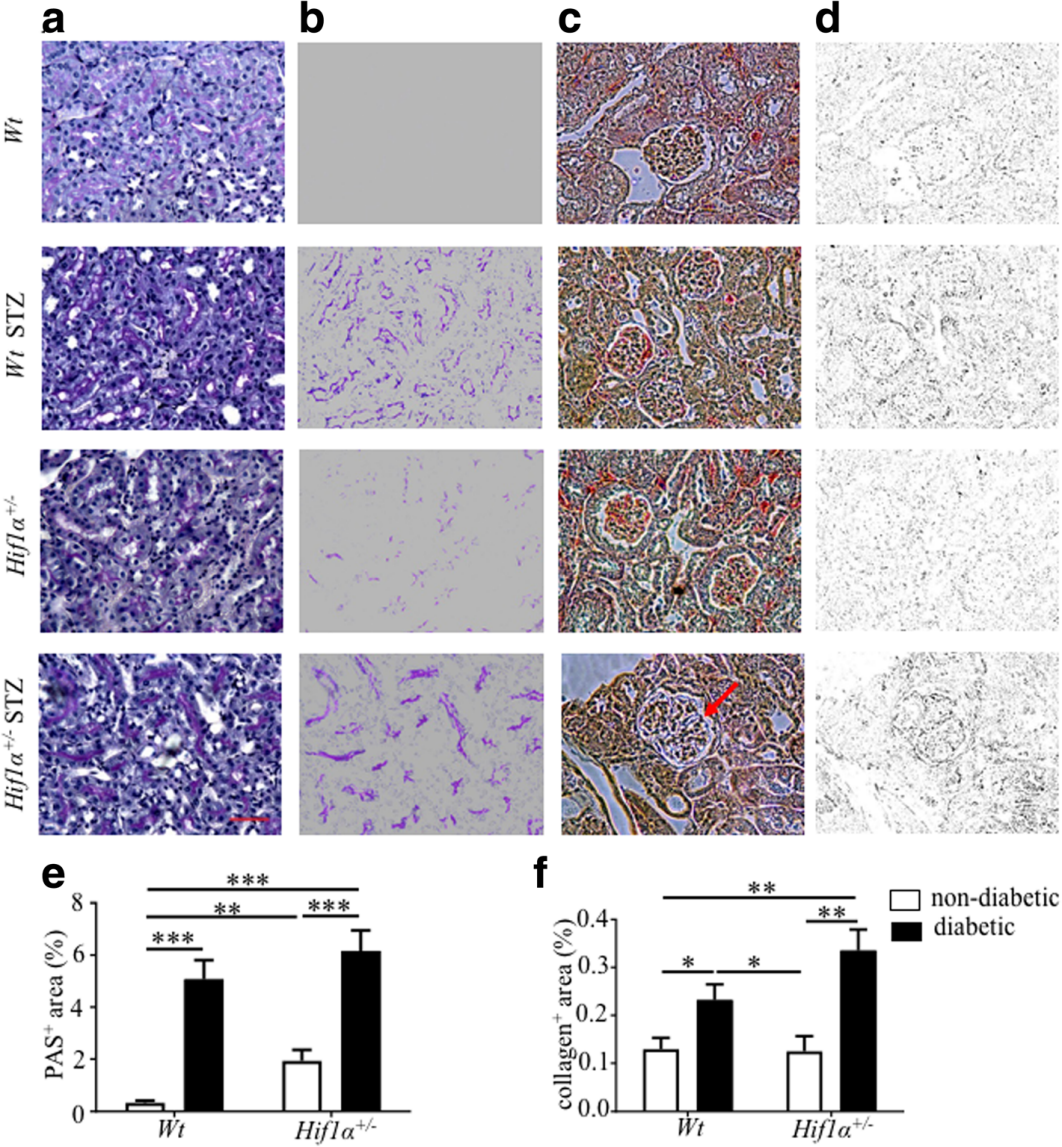
PAS and trichrome-staining of the renal cortex in non-diabetic and diabetic *Wt* and *Hif1α* ^*+/−*^ mice. **a** Representative PAS staining of 8 μm kidney sections showed advanced glycation end products in diabetic *Wt* (*Wt* STZ) and diabetic *Hif1α* ^*+/−*^ (*Hif1α* ^*+/−*^ STZ). The strongest positive staining was detected in the tubular part of the kidney section. **b** Delineated PAS^+^ area in the kidney section using Adobe Photoshop. **c** Representative Masson’s trichrome-staining of 8 μm kidney sections showed increased fibrosis with increased collagen fibers in the renal cortex of diabetic kidneys. **d** Delineated collagen^+^ area in the kidney section by ImageJ. **e**-**f** A relative quantification of staining was determined as a percentage of positive area in the field of view by ImageJ. Scale bar 100 μm. The values represent means ± SEM (*n* = 3 sections/3 samples/group). Statistical significance differences were tested by two-way ANOVA (diabetes effect: PAS and trichrome (*P* < 0.0001); effect of genotype in PAS staining (*P* = 0.04)). *Significant differences by post hoc pairwise comparison, *P < 0.05, **P < 0.01, ***P < 0.0001

Periodic acid–Schiff (PAS) staining is a method for the detection of AGE, non-enzymatic tissue modifications [26]. A weak positive staining was detected in the tubular part of the renal cortex of non-diabetic *Hif1α* ^*+/−*^ mice (Fig. 2a, b), suggesting AGE modifications due to *Hif1α* deficiency even under normal conditions. A significantly higher production of AGE products was detected in both the diabetic *Wt* and *Hif1α* ^*+/−*^ renal cortex (Fig. 2e). Using Masson’s trichrome staining, we analyzed interstitial collagen deposition in the renal cortex as an index of interstitial fibrosis and overall tissue remodeling (Fig. 2c, d). We did not detect any differences in collagen deposition between non-diabetic *Wt* and *Hif1α* ^*+/−*^. Collagen accumulation was significantly increased in both *Wt* and *Hif1α* ^*+/−*^ diabetic groups, although the trend of more abundant tissue remodeling was evident in the diabetic *Hif1α* ^*+/−*^ renal cortex (Fig. 2f). Based on light microscopy evaluation, we detected only mild mesangial expansion without nodular sclerosis (Fig. 2c, arrow), classified as class I/II DN, which is a characteristic early stage of DN [27].

### Molecular changes in the renal cortex of diabetic mice with *Hif1α*^*+/−*^ deficiency

**Fig. 3:**
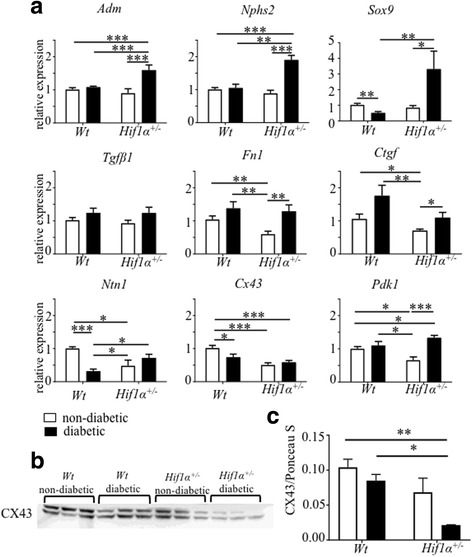
Gene expression changes in the renal cortex of diabetic and non-diabetic *Wt* and *Hif1α* ^*+/−*^ mice. **a** The relative gene expression changes were analyzed using RT-qPCR and quantified with ΔΔCT method. The values represent means ± SEM (*n* = 8 samples/group). Statistical significance differences in normalized Ct values were tested by two-way ANOVA followed by post hoc pairwise comparison tests *P < 0.05, **P < 0.01, ***P < 0.001*.* **b** The renal cortex extracts were prepared and probed on Western blots with antibodies directed against connexin 43 (CX43; ~43 kDa). Representative immunoblot for CX43 is shown. **c** Combined results obtained by densitometric evaluation of the Western blots of CX43 using cortical extracts from three mice per group. Ponceau S staining was used as the loading control. Data were analyzed by ImageJ software. Statistical significance differences were tested by two-way ANOVA followed by post hoc pairwise comparison tests *P < 0.05, **P < 0.01*.* Abbreviations: adrenomedullin (*Adm*), podocin *(Nphs2),* SRY (Sex Determining Region Y)-Box 9 *(Sox9)),* transforming growth factor beta 1 *(Tgfβ1),* fibronectin 1 *(Fn1),* connective tissue growth factor (*Ctgf*), netrin *(Ntn1),* connexin 43 *(Cx43),* and pyruvatdehydrogenase kinase1 *(Pdk1)*

This study assesses the effects of partial *Hif1α* ^*+/−*^ deficiency on the progression of DN in early stages of diabetes. We analyzed the expression of genes associated with extracellular matrix expansion, podocyte dysfunction, and profibrotic responses with a specific focus on HIF-1*α* direct target genes (Fig. 3a). *Hif1α* partial deficiency was demonstrated by a reduced expression of HIF-1-targeted genes (*Pdk1, Ntn1, Ctgf,* and *Fn1)* in the renal cortex under non-diabetic conditions. The mRNA level of adrenomedullin (*Adm),* a potent vasodilatory peptide hormone, was increased in the diabetic *Hif1α* ^*+/−*^ kidney cortex compared to other experimental groups. The relative gene expression of podocin (*Nphs2*), a marker for podocyte damage, was significantly elevated only in the diabetes-exposed *Hif1α* ^*+/−*^ renal cortex. Collagen accumulation has been associated with the up-regulation of the transcription factor *Sox9,* a direct HIF-1*α* target [28]. A significant increase in the mRNA level of the *Sox9* gene was detected in the diabetic *Hif1α* ^*+/−*^ kidney cortex. A pivotal cytokine in the profibrotic responses [29], transforming growth factor beta 1 (*Tgfβ1*)*,* was significantly increased in both diabetic groups (the effect of diabetes, *P* < 0.05 by two-way ANOVA). Fibronectin (*Fn1*) and connective tissue growth factor (*Ctgf*), classical markers of fibrosis and indicators of extracellular matrix accumulation, were increased in diabetic mice (significant effect of diabetes *P* < 0.008 and effect of genotype *P* < 0.01 by two-way ANOVA). As HIF-1*α* direct target genes, both *Fn1* and *Ctgf* were significantly decreased in non-diabetic *Hif1α* ^*+/−*^ mice, suggesting impaired HIF-1α regulation. The partial deficiency of netrin-1 (*Ntn1*) results in kidney microvascular dysfunction and accelerated DN [30]. Correspondingly, the expression of *Ntn1* was reduced in the diabetic *Wt* and *Hif1α* ^*+/−*^ renal cortex compared with non-diabetic *Wt*. We also found a significant reduction of *Ntn1,* a direct HIF-1*α* target*,* in the non-diabetic *Hif1α* ^*+/−*^ compared to *Wt* mice, indicating altered HIF-1*α* regulation. We found that the expression of *Cx43* [31] in the renal cortex was significantly attenuated in non-diabetic *Hif1α* ^*+/−*^. Cx43 participates in intercellular communication and is down-regulated by diabetes [32]. Accordingly, in our experimental diabetic model, both diabetic groups *Wt* and *Hif1α* ^*+/−*^ showed decreased *Cx43* expression compared to non-diabetic *Wt*. Consistently, decreased protein levels of CX43 were detected in the renal cortex of diabetic *Hif1α* ^*+/−*^ mice, indicating impaired intercellular communication that may cause endothelial cell dysfunction and glomerular injury (Fig. 3b, c).

### Podocyte dysfunction in diabetic *Hif1α*^*+/−*^ mice

**Fig. 4:**
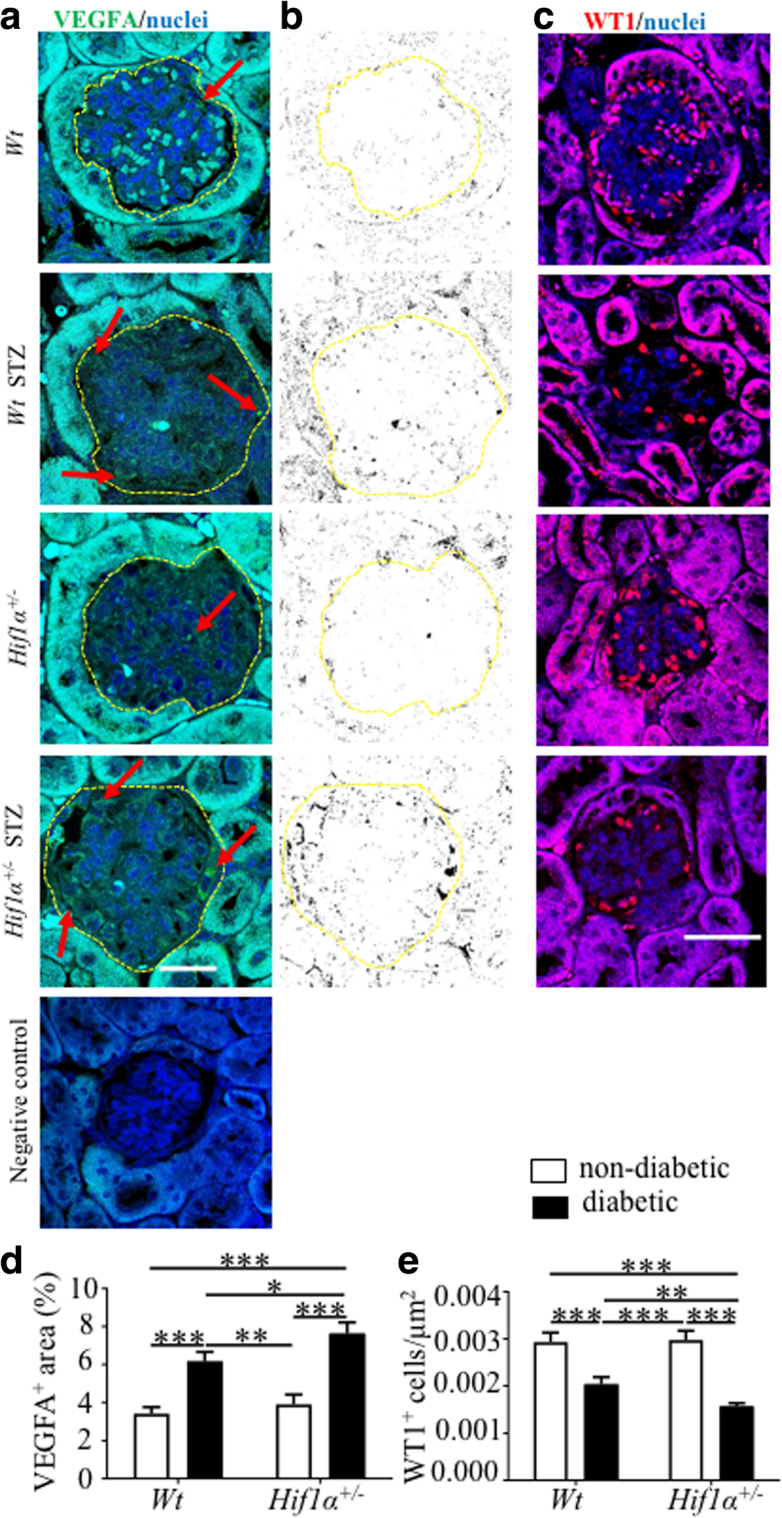
Molecular changes in the glomerulus of *Wt* and *Hif1α* ^*+/−*^ mice. **a** Representative confocal microscopy images of 8 μm sections of the glomerulus stained with anti-VEGFA antibody (*green*) show the highest expression of VEGFA in diabetic *Hif1α* ^*+/−*^ kidneys. The area of the glomerulus is outlined by the yellow dashed line. Arrows indicate VEGFA expression (*sharp green*) in the glomerulus. Negative control is without primary antibody. Images are stacked Z-plane sections; nuclei are counterstained with Hoechst 33,342 (*blue*); scale bar 20 μm. **b** Delineated VEGFA^+^ area by ImageJ. **c** Staining of mature and fully functional podocytes with anti-WT1 antibody (*red*) shows the largest loss of podocytes in the diabetic *Hif1α* ^*+/−*^ renal cortex; nuclei are counterstained with Hoechst 33,342 (*blue*); scale bar 50 μm. **d** Relative quantification of VEGFA expression was determined as a percentage of VEGFA^+^ area per glomerular area. **e** Quantification of podocyte density as a number of WT1^+^ cells per glomerular area. The values represent means ± SEM (*n* = 10 glomerulus/3 sections/3 mice/group). Two-way ANOVA detected statistical significance of diabetes effect (*P* < 0.0001) followed by post hoc pairwise comparison tests *P < 0.05, **P < 0.01, ***P < 0.001. Abbreviations: vascular endothelial growth factor (VEGFA*),* Wilms tumor 1 (WT1)

Podocytes highly express vascular endothelial growth factor (VEGFA) and any small changes in VEGFA levels cause significant aberrations in glomerular structure [33]. We detected significantly higher VEGFA expression in the glomerulus of diabetic *Hif1α* ^*+/−*^ mice compared to diabetic *Wt* (Fig. 4a–d), indicating early deleterious changes in diabetic disease. Podocyte loss was also demonstrated by staining with WT1 (Fig. 4c), a nuclear marker of mature and fully functional podocytes [34]. The number of WT1 positive podocytes per glomerulus area was significantly decreased in diabetic *Hif1α* ^*+/−*^ compared to diabetic *Wt* (Fig. 4e). Together with the increased expression *Nphs2* (Fig. 3a), a marker for podocyte damage, our data thus indicate that *Hif1α* partial deficiency combined with diabetes accelerates podocyte loss and the inability to sustain the glomerular filtration barrier.

### Diabetes-induced changes associated with profibrotic processes and accumulation of extracellular matrix

**Fig. 5:**
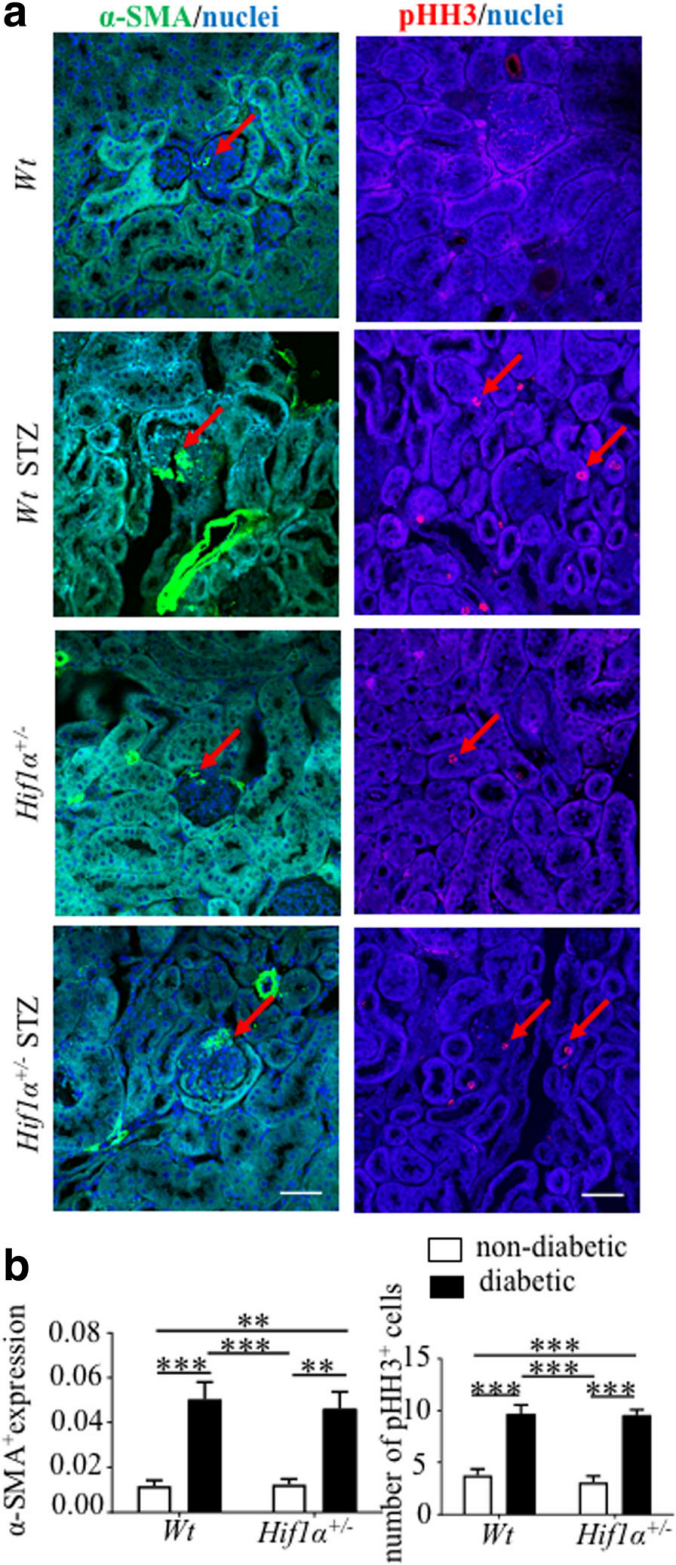
Diabetes-induced changes in α-SMA and pHH3 expression in the kidney of *Wt* and *Hif1α* ^*+/−*^ mice. **a** Confocal imaging of 8 μm sections of the kidney stained with anti-α-SMA and anti-pHH3 antibodies. Images are stacked Z-plane sections; nuclei are counterstained with Hoechst 33,342 (*blue*); scale bars 50 μm. **b** Quantification of α-SMA^+^ area was done per glomerular area (10 glomerulus/each section) and the positive pHH3^+^ staining was determined as a number of the positive nuclei in the field of view of the renal cortex. The values represent means ± SEM (*n* = 3 sections/3mice/group). Statistical significance assessed by two-way ANOVA: diabetes effect in the expression of pHH3 and α-SMA (*P* < 0.0001). Post hoc pairwise comparison **P < 0.01, ***P < 0.001. Abbreviations: alpha smooth muscle actin (α-SMA), phospho-histone H3 (pHH3)

Our gene expression profiling analyses showed significant changes in the expression of profibrotic markers induced by the diabetic milieu. We analyzed the expression of α-SMA, an excellent prognostic indicator of renal fibrosis progression and marker of extracellular matrix accumulation [35]. The expression of α-SMA was significantly increased in both diabetic *Wt* and *Hif1α* ^*+/−*^ mice (Fig. 5a, b). The diabetic milieu triggers early tubular cell proliferation. Proximal tubule growth involves an early period of hyperplasia followed by a shift to hypertrophy [24]. Consistent with a hyperplasia phenotype in an early stage of DN, the number of mitotic cells found in tubular cells was increased in both diabetic *Wt* and *Hif1α* ^*+/−*^ kidneys in comparison with non-diabetics (Fig. 5a, b). We did not detect any significant differences associated with the *Hif1α* ^*+/−*^ phenotype.

## Discussion

Our partial deficiency *Hif1α* model provides the first model that tests in vivo the function of HIF-1α in the development and progression of diabetes-induced renal damage. Previous work has only provided indirect evidence for the role of HIF-1α, using a HIF-1 inhibitor [6] or HIF-1 activator [16]. Our data extend previous findings that HIF-1α signaling is activated in the kidneys of experimental models with type I and type II diabetes and that it may be relevant to the development of DN [4, 5, 12]. We examined the role of HIF-1α in the early stage of disease using the STZ-induced diabetic mouse model characterized by hyperglycemia (blood glucose levels > 13.9 mmol/L) and insulinopenia. We found that *Hif1α* partial deficiency significantly accelerated the manifestation of pathological changes associated with the progression of DN. Changes in serum biochemical parameters associated with diabetic glomerular injury and progression of chronic kidney disease were more significant in diabetic *Hif1α* ^*+/−*^ compared to diabetic *Wt* mice. The combination of *Hif1α* deficiency and diabetes resulted in an altered transcriptional expression profile of the renal cortex and decreased survival of podocytes.

Hypoxia represents an early and potentially initiating factor in the development and progression of chronic kidney diseases including DN [4, 36]. HIF-1 mediates hypoxia-induced cellular responses through the regulation of genes involved in cell metabolism, glucose utilization, angiogenesis, oxidative stress, apoptosis, and proliferation. However, the activation of HIF-1 in the diabetic kidney may be suboptimal despite profound renal hypoxia, as suggested by a large body of evidence showing that the diabetic milieu deregulates the HIF-1α pathway [13–15]. In recent years, HIF-1α genetic polymorphisms have emerged as potentially important determinants of disease severity and adverse outcomes [37, 38]. Nonetheless, given the diversity of HIF-1 signaling, it remains controversial whether the activation of HIF-1 signaling exerts a beneficial or harmful role in the progression of renal diseases, particularly DN.

Persistent, chronic exposure to hypoxia is associated with structural tissue remodeling, such as renal fibrosis, inflammation, apoptosis and loss of microvasculature. HIF-1 signaling is an important protective physiological mechanism activated to counteract hypoxia and prevent renal damage (for review, see [39]). For example, the global inactivation of the *Vhlh* gene by the Cre-loxP system resulted in HIF-1α and HIF-2α stabilization and suppressed fibrogenesis in mice subjected to unilateral ureteral obstruction [40]. Other studies using pharmacological approaches for systemic HIF-1 activation demonstrated improved proteinuria and histological parameters in experimental chronic kidney disease models [41, 42]. In contrast, other studies have shown that sustained HIF-1 activation may have unfavorable effects. Genetic inactivation of the *Vhlh* gene in tubular epithelial cells resulted in constitutive HIF-1α stabilization and accelerated renal fibrosis [43]. Similarly, the genetic ablation of *Hif1α* in the renal proximal tubule inhibited tubulointerstitial fibrosis in the in vivo model of unilateral ureteral obstruction [44]. These data suggest that HIF-1α may play different roles in the progression of chronic kidney diseases depending on the mode of activation, cell-type specific action, and local versus global HIF-1α stabilization. Thus, these conflicting results reflect the complexity of the adaptive responses mediated by HIF-1.

Similar discrepancies have been reported regarding the role of HIF-1 in DN. An indirect approach using YC-1 [3-(5′-hydroxymethyl-2′-furyl)-1-benzyl indazole], a HIF-1 inhibitor, reduced glomerular hypertrophy and AGE in the type 1 diabetes mouse model [6]. In contrast, an induction of HIF-1α by CoCl_2_ reduced proteinuria and histological markers of kidney injury in an obese type 2 diabetes model [16] and in STZ-induced DN in rats [3]. In conjunction with these studies, our data demonstrate that a partial *Hif1α* deficiency promotes the diabetes-induced kidney injury. *Hif1α* partial deficiency was associated with a reduced expression of HIF-1-targeted genes *Pdk1, Ntn1, Ctgf,* and *Fn1*. Serum glucose levels were significantly increased in *Hif1α* ^*+/−*^ mice compared to *Wt*, implying systemic changes in glucose metabolism in association with *Hif1α* partial deletion, which may contribute to the enhanced pathogenesis. HIF-1, by regulating the expression of glucose transporter GLUT1 and glycolytic enzymes, affects glucose homeostasis, including the regulation of glucose-stimulated insulin secretion (GSIS) from the pancreatic beta-cells [45]. Targeted disruption of *Hif1α* in pancreatic beta-cells resulted in glucose intolerance, impaired GSIS, and beta-cell dysfunction [46]. Thus, the increased serum glucose levels in our diabetic *Hif1α* ^*+/−*^ mice were in accordance with the changes in beta-cell function and impaired glucose homeostasis.

These changes were accompanied by glomerular damage, as indicated by a significant loss of podocytes and increased expression of podocin, a marker for podocyte damage, in the diabetic *Hif1α* ^*+/−*^ renal cortex. These results suggest that HIF-1α functional impairment affected the survival of podocytes in the diabetes-exposed kidney. It is important to notice that systemic pharmacological approaches used in previous studies of DN [3, 6, 16] may produce HIF-1-independent effects and may also affect other tissues resulting in different responses in diabetes-exposed kidneys.

In response to injury, mesangial cells transdifferentiate and synthesize different extracellular matrix proteins, which is an important pathological event during glomerulosclerosis and the progression of DN. The increased expression of transcription factor SOX9 has been associated with changes in mesangial cells and expansion of the mesangial area in the progression of DN [28]. Additionally, the activation of SOX9 is critical for the early damage and repair response of injured renal tubule cells [47]. This repair response in the chronically active form may represent an additional mechanism triggering long-term pathological responses resulting in kidney damage. Not only HIF-1 mediates *Sox9* expression, ERK1/2 signaling [48] or BMP4 [28] may also induce *Sox9* expression. Furthermore, advanced glycation end products (AGEs) have been shown to induce *Sox9* expression [28]. Thus, we can postulate that increased *Sox9* expression in the diabetic *Hif1α* ^*+/−*^ renal cortex may indicate a) an early transcriptional response to renal injury or/and b) regulatory compensatory response to *Hif1α* deficiency and diabetic environment.

We found increased collagen accumulation in both diabetic *Hif1α* ^*+/−*^ and *Wt* mice. Correspondingly, the expression of markers of fibrosis and extracellular matrix accumulation, *Tgfβ1,* fibronectin, *Ctgf*, and α-SMA were increased in both diabetic *Hif1α* ^*+/−*^ and *Wt* mice. These results indicate that fibrosis in the diabetic kidney was not affected by the global reduction of *Hif1α*, at least not in the early phase of diabetic exposure. In line with our observations are studies where the global *Hif1α* deletion using the *Ubc-cre/ERT2* system did not affect collagen accumulation, although inflammation and renal injury were enhanced by *Hif1α* deletion in the model of unilateral ureteral obstruction [49].

VEGFA stimulates endothelial cell proliferation and has a key role in physiologic and pathologic angiogenesis in different tissues. In the kidney, VEGFA regulates glomerular permeability and maintenance of the glomerular tuft, and overall maintenance of kidney integrity [50]. VEGFA is tightly regulated as shown by glomerular-selective overexpression or deletion of VEGFA resulting in severe and early renal pathologies [33]. Renal diseases are frequently associated with impaired angiogenesis, capillary loss, and a reduction of VEGFA expression. In contrast, in diabetic nephropathy, renal VEGFA levels are elevated in experimental models as well as in diabetic patients [51–53] The upregulation of VEGFA has been proposed as a contributing mechanism to renal dysfunction during the early phase of diabetes [53, 54]. Inhibition of VEGFA at the onset of diabetes abolished the associated diabetes-glomerular hyperfiltration, glomerular hypertrophy, and urinary albumin excretion in the type I diabetes model [53]. In our study, consistent with previously published data, VEGFA expression was significantly increased in the glomerulus of diabetic *Hif1α* ^*+/−*^ compared to the diabetic *Wt*, indicating a faster progression of renal dysfunction in diabetes (Fig. 4). The cause of the upregulation of VEGFA in the diabetic kidney remains speculative; however, multiple factors may be implicated [53]. Renal dysfunction of diabetic *Hif1α* ^*+/−*^ mice was further supported by the increased expression of *Adm* in the diabetic *Hif1α* ^*+/−*^ renal cortex. The upregulation of *Adm*, which encodes a potent vasorelaxant peptide, is associated with glomerular hyperfiltration and dilatation of the glomerular capillaries in the acute phase of type 1 diabetes [55]. Notably, serum albumin levels were significantly decreased in diabetic *Hif1α* ^*+/−*^ mice (Fig.1).

A limitation of our study is the global nature of the *Hif1α* deletion. We are unable to determine which cell type or which combinations of cell types are contributing to the increased susceptibility of *Hif1α* ^*+/−*^ mice to DN. The global deletion of *Hif1α* may affect other tissues and it may indirectly escalate pathological functional and structural changes in the kidney of *Hif1α* ^*+/−*^ mutants. At the same time, our model reproduces the conditions of a global inhibition of HIF-1 signaling, such as in pharmacological targeted-HIF-1 inhibition.

## Conclusions

Taken together, our studies point to a protective role of HIF-1 signaling in the early phase of adaptive responses to diabetic environment and that impaired HIF-1 signaling results in a faster progression of DN. Furthermore, our data suggest a potential role of *Hif1α* genetic variations in the manifestation of DN. Although the modulation of HIF-1 activity is a high-priority target for clinical therapies, our data accentuate the necessity of optimizing possible pharmacological inhibition of HIF-1 in therapeutic applications for the treatment of DN.

## Additional file

Additional file 1: Table S1. The additional file lists primer sequences for genes analyzed by qPCR. (JPEG 122 kb)

## Abbreviations

Adm: Adrenomedullin
AGE: Advanced glycation end products
Ctgf: Connective tissue growth factor
Cx43: Connexin 43
DN: Diabetic nephropathy
Fn1: Fibronectin 1
GSIS: Glucose-stimulated insulin secretion
HIF-1: Hypoxia inducible factor 1
Nphs2: Podocin
Ntn1: Netrin
PAS: Periodic acid–Schiff staining
Pdk1: Pyruvatdehydrogenase kinase1
ROS: Reactive oxygen species
Sox9: SRY (Sex Determining Region Y)-Box 9
STZ: Streptozotocin
Tgfβ1: Transforming growth factor beta 1
Vegfa: Vascular endothelial growth factor
Wt: Wild-type
Wt1: Wilms tumor 1 homolog
α-SMA: Alpha 2 smooth muscle actin
