# Supplementary figures and images for: Renal injury is accelerated by global hypoxia-inducible factor 1 alpha deficiency in a mouse model of STZ-induced diabetes
Source: BMC Endocr Disord. 2017 Aug 3;17:48. doi: 10.1186/s12902-017-0200-8 (PMC5543752; doi:10.1186/s12902-017-0200-8)

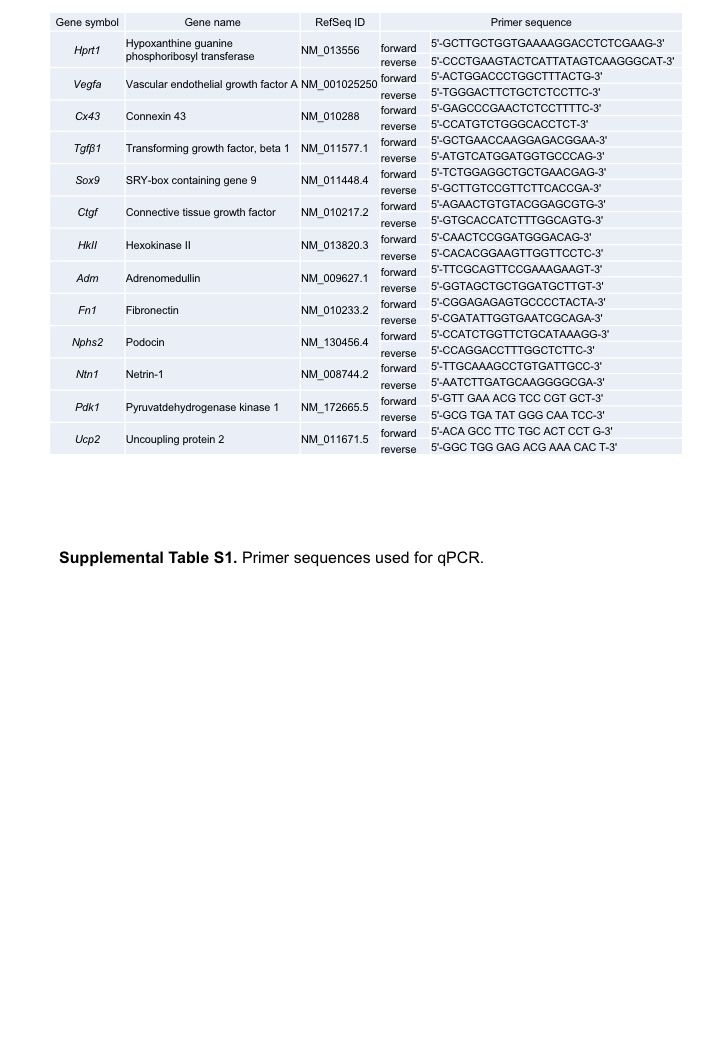

Supplement: Additional file 1: Table S1. — The additional file lists primer sequences for genes analyzed by qPCR. (JPEG 122 kb) [file 12902_2017_200_MOESM1_ESM.jpg]
